# A CONTENT ANALYSIS OF CHILDREN’S LITERATURE ON DEATH AND DYING

**DOI:** 10.1093/geroni/igae098.3056

**Published:** 2024-12-31

**Authors:** Celine Manternach, Hallory Domnick

**Affiliations:** University of Northern Iowa, Cedar Falls, Iowa, United States; University of Northern Iowa, Cedar Falls, Iowa, United States

## Abstract

The purpose of this project was to analyze children’s literature on death and dying published between 2014-today. This project builds on previous research published prior to 2014. N = 40 books were reviewed for the following content: Biological facts including type of loss, emotional responses, socio-cultural practices and beliefs, and the number of euphemisms used. Books available from local libraries and Amazon were used for this project. Examples from each book were included in Excel and frequencies and percentages were summed after analysis. A total of 10 (25%) books included biological facts, such as “heartbeat stops,” 18 (45%) included emotional responses such as “Dad’s eyes were red,” and 8 (20%) included socio-cultural practices and beliefs such as “we all have beliefs and we don’t know what happens when we die.”A total of 5(8%) books included euphemistic terms such as “gone to a better place” for death. Overall, findings demonstrated that authors tended to write about emotional responses related to grief most often, whereas fewer wrote about socio-cultural practices and beliefs. Given the diversity and complexity of practices and beliefs on death and dying, additional children’s books published in this area are needed to capture the diversity of experiences. While explaining death to children can be challenging, literature is a tool that parents can use to guide the conversation. However, not all children’s books are created with the same intent, and it is important for adults to pre-screen books to determine how appropriate the book is for their child.

